# Thermal resilience may shape population abundance of two sympatric congeneric *Cotesia* species (Hymenoptera: Braconidae)

**DOI:** 10.1371/journal.pone.0191840

**Published:** 2018-02-13

**Authors:** Reyard Mutamiswa, Honest Machekano, Frank Chidawanyika, Casper Nyamukondiwa

**Affiliations:** 1 Department of Biology and Biotechnological Sciences, Botswana International University of Science and Technology (BIUST), Palapye, Botswana; 2 Agricultural Research Council, Plant Protection Research, Weeds Division, Hilton, South Africa; 3 School of Lifesciences, University of KwaZulu-Natal, Pietermaritzburg, South Africa; Universite du Quebec a Chicoutimi, CANADA

## Abstract

Basal and plasticity of thermal tolerance determine abundance, biogeographical patterns and activity of insects over spatial and temporal scales. For coexisting stemborer parasitoids, offering synergistic impact for biological control, mismatches in thermal tolerance may influence their ultimate impact in biocontrol programs under climate variability. Using laboratory-reared congeneric parasitoid species *Cotesia sesamiae* Cameron and *Cotesia flavipes* Cameron (Hymenoptera: Braconidae), we examined basal thermal tolerance to understand potential impact of climate variability on their survival and limits to activity. We measured upper- and lower -lethal temperatures (ULTs and LLTs), critical thermal limits [CTLs] (CT_min_ and CT_max_), supercooling points (SCPs), chill-coma recovery time (CCRT) and heat knock-down time (HKDT) of adults. Results showed LLTs ranging -5 to 5°C and -15 to -1°C whilst ULTs ranged 35 to 42°C and 37 to 44°C for *C*. *sesamiae* and *C*. *flavipes* respectively. *Cotesia flavipes* had significantly higher heat tolerance (measured as CT_max_), as well as cold tolerance (measured as CT_min_) relative to *C*. *sesamiae* (*P*<0.0001). While SCPs did not vary significantly (*P*>0.05), *C*. *flavipes* recovered significantly faster following chill-coma and had higher HKDT compared to *C*. *sesamiae*. The results suggest marked differential basal thermal tolerance responses between the two congeners, with *C*. *flavipes* having an advantage at both temperature extremes. Thus, under predicted climate change, the two species may differ in phenologies and biogeography with consequences on their efficacy as biological control agents. These results may assist in predicting spatio-temporal activity patterns which can be used in integrated pest management programs under climate variability.

## 1. Introduction

Abiotic factors such as temperature and relative humidity have direct effects on development, reproduction, abundance, biogeography [1,2] and survival of ectotherms [3], including parasitoids [4]. Of these, temperature is considered the predominant abiotic factor affecting both herbivorous insects and their antagonistic biological control agents [5,6]. In biological control programs of insect pests, success can be mediated by the parasitoids`responses to environmental variation including temperature and precipitation [6,7]. In agroecosystems, long evolved relationships between the herbivorous insects and their parasitoid species can be perturbed under unfavourable environmental conditions resulting in temporal asynchrony of multi-trophic level communities through changes in population dynamics and biogeography [4]. Given that most insects are ectotherms, and their population growth is temperature driven, extreme temperatures have a bearing on their rate of development as well as biochemical and physiological processes [4,8,2]. Since most insects have a limited ability to control body temperature, they have developed a range of mechanisms for survival under stressful thermal environments [9]. Some of the mechanisms involve behavioral avoidance [9], morphological adaptations as well as daily [10] and seasonal [11] thermal tolerance adjustment. However, failure to employ some compensatory mechanisms to survive these extreme temperatures may offset fitness traits, hence limited activity and poor performance of life-history traits ultimately leading to population decline and seldom species extinction [12].

Insect temperature tolerance is typically not static [13], and may be influenced by a range of factors including age [8], developmental stage [14] thermal history [15,16] and species ecological and evolutionary history (e.g. temperate vs. tropical environment) [17]. These factors, combined with complex interactions between duration and severity of exposure may determine an insect’s thermal tolerance with longer or more severe exposures typically lethal [13]. Thermal history and physiological tolerances vary in insects with some species emerging ‘winners’ whilst others succumb to detrimental effects of climate change in the short term [18] thus affecting their population dynamics and community-wide interactions [7]. Hence, for congeneric sympatric species with of the same trophic level, environmental variability may differentially impact species through variable limitations on survival or longevity and key activities such as dispersal, reproduction and diapause [19]. For example, thermal tolerance measured on two closely related sympatric species, *Aphidius avenae* Haliday and *Aphidius rhopalosiphi* De Stefani-Perez (Hymenoptera: Aphidiinae) matched their different seasonal activities thereby confirming the importance of temperature tolerance on parasitoid population dynamics [20].

Parasitoids such as *Cotesia sesamiae* Cameron and *Cotesia flavipes* Cameron (Hymenoptera: Braconidae) have different evolutionary and thermal history, even though they have been reported to coexist occupying the same ecological niche [21,22]. This competitive interaction has been beneficial in African agriculture as the combined effect of the two species has led to increased pest suppression [23]. However of concern, is how population decrease in one parasitoid species following environmental perturbations may affect host stemborer outbreaks, and the likely costs of increased pest pressure.

*Cotesia flavipes* is a gregarious larval endoparasitoid of lepidopteran stemborers [24]. It is native to Asia and is the closest coevolved larval parasitoid of *Chilo partellus* Swinhoe (Lepidoptera: Crambidae) [25]. Following its first release in Kenya in 1993 [25], it was subsequently introduced in various east and southern African countries where it successfully controlled *C*. *partellus* in maize and sorghum [26]. On the other hand, *C*. *sesamiae*, is indigenous to Africa and endemic to the sub-Saharan region. It belongs to the *C*. *flavipes* monophyletic complex [27] and is a generalist gregarious endoparasitoid of the entire lepidopteran cereal stemborer complex [28]. Morphologically, *C*. *sesamiae* and *C*. *flavipes* adults are similar and both attack medium to large sized larvae of lepidopteran cereal stemborers [29]. These parasitoid species have a short generation time lasting 18 days at 25–30°C [30]. Although multiple parasitism has been reported on *C*. *partellus* and *S*. *calamistis*, *C*. *flavipes* has a higher host searching capacity and attacks more larvae than *C*. *sesamiae* when *C*. *partellus* is the host [31]. *Cotesia flavipes* and *C*. *sesamiae* have been reported to reduce lepidopteran stemborer densities between 32 and 55% in agroecosystems and are of importance in cereal farming in sub-Saharan Africa [23], in particular, maize (*Zea mays* L.) and sorghum *Sorghum bicolor* (L.) Moench which are the staple crops in large parts of this region. The distribution of these two *Cotesia* species is influenced by climate, with *C*. *sesamiae* common in wetter regions and *C*. *flavipes* common in warm and dry regions [28].

Due to the discrepancies in both evolutionary origin and habitat preferences among these parasitoids, we sought to investigate survival and functional activity limits under acute thermal variability, typical in African agroecosystems. With global mean temperatures projected to increase by 1.4 to 5.8°C by 2100 [32], coupled with increased frequencies of heat waves and cold snaps [33], a comprehensive understanding of the thermal tolerance and performance of parasitoid species [4,34] is of paramount importance for effective prediction of climate change impacts on insect population dynamics, extinction [35] and efficacy of biological control [36]. Previous studies reported that climate change may interrupt host-parasitoid relationships due to differences in thermal preferences thus increasing the risk of host outbreaks [4,18] While several studies have assayed thermal tolerance in insect pests [8,11,2], coexisting parasitoid species [37] and recently *C*. *partellus* and its parasitoid *C*. *sesamiae* [18], to our knowledge, no studies have investigated the thermal tolerance of coexisting lepidopteran stemborer parasitoids of importance in agriculture. Here, we investigated basal thermal tolerance of indigenous and exotic larval endoparasitoids (*C*. *sesamiae* and *C*. *flavipes*, respectively) of lepidopteran cereal stemborers and discuss its potential implications on geographic distribution and biological control under climate change. We investigated ability to tolerate extreme temperatures (lower and upper) following exposure to different ranges of temperature-time interactions, lower and upper critical limits to activity, supercooling points, time to heat knock-down and recovery following chill coma. Since *C*. *sesamiae* is predominant in warm wetter regions, indigenous to Africa and attacks a wide range of stemborer species [38,28], we hypothesised that it has a higher basal thermal tolerance than the exotic *C*. *flavipes*. An in-depth understanding of the species`basal thermal tolerance is of importance in pest management including site selection for initial parasitoid release (based on thermal preferences), timing of augmentative releases and general designing of efficacious biological control programs for lepidopteran stemborers in changing climate. The study is also significant in explaining how introduced exotic parasitoids in classical biological control may shape ecosystem function and competitive ability of congeneric species occupying the same niche under dynamic environmental conditions.

## 2. Materials and methods

### 2.1 Study organisms and rearing conditions

Initial colonies of *C*. *sesamiae* and *C*. *flavipes* were obtained from South African Sugarcane Research Institute (SASRI), South Africa and International Centre for Insect Physiology and Ecology (ICIPE), Kenya, respectively. These insects had been in the laboratory for more than 20 generations with regular supplementation with wild populations to maintain heterozygosity. Because of variations in host preferences, *Cotesia sesamiae* colony was created using parasitized *Sesamia calamistis* larvae whilst parasitised *C*. *partellus* larvae where used for *C*. *flavipes* colony. Both colonies were maintained in the climate chambers (HPP 260, Memmert GmbH + Co.KG, Germany) under 12:12 day and night photocycles, 28 ± 1°C and 65 ± 10% RH on artificial diet [39] in 30 ml plastic vials with perforated screw-cap lids. Emerging parasitoid cocoons were separated and held according to species, under similar conditions in open Petri dishes placed in Bugdorm rearing cages (240mm^3^; Bugdorm-BD43030F, Megaview Science Co., Ltd, Taiwan) until eclosion. Eclosed parasitoids had access to food *ad libitum* (25% honey: water from a cotton wick) until they were used in thermal tolerance assays as 24–48 h-old adults.

### 2.2 Lower and upper lethal temperature assays

Using a direct plunge protocol in programmable water baths (Systronix, Scientific, South Africa), containing a mixture of propylene glycol and water (1:1 ratio to enable sub-zero temperature operation without freezing), LLT and ULT were assayed using established protocols by Terblanche et al. [40], Chidawanyika and Terblanche [11], Mutamiswa et al. [18]). In brief, ten 24–48 h old adults replicated five times for each species (*C*. *sesamiae* and *C*. *flavipes*) (n = 50) were placed in 30ml polypropylene vials with perforated screw-cap lids and placed in water tight zip-lock bag which was submerged in a programmable water bath for each temperature/time treatment for either ULT or LLT assays. Digital thermometers (Fluke 53/54IIB, Fluke Cooperation, USA) were used to monitor the temperature of the water bath for the duration of each treatment. Post treatment, propylene vials containing assayed insects were placed in a climate chamber (28 ± 1°C, 65 ± 10% RH and 12:12 day and night photocycles, and supplied with food (25% honey: water from a cotton wick) during the whole recovery period. Survival was then recorded 24 hours post treatment. For the purposes of this study, survival was defined as coordinated muscle response to stimuli such as gentle prodding, or normal behavior such as flying (24 h post treatment). ULT and LLT assays for both species ranged from 35 to 44°C and -15 to 5°C respectively at 0.5, 1, 2 and 4 h duration of exposure until 0–100% mortality was recorded. In all the assays 24–48 h-old adult parasitoids of mixed sex were used, since sex seem to play no significant role in thermal tolerance traits (see [8,41]).

### 2.3 Critical thermal limits (CTLs)

Critical thermal limits (CT_min_ and CT_max_) were measured using a dynamic protocol as outlined by Nyamukondiwa and Terblanche [8]. Ten individual adults of *C*. *sesamiae* or *C*. *flavipes* (24–48 h old) were randomly placed in a series of insulated double-jacketed chambers (‘organ pipes’) connected to a programmable water bath (Lauda Eco Gold, Lauda DR.R. Wobser GMBH and Co. KG, Germany) filled with 1:1 water: propylene glycol and subjected to a constant cooling or heating rate. In the ‘organ pipes’, insects were first given 10 minutes to equilibrate at 28°C (equivalent to the benign rearing temperature) before ramping temperature up (CT_max_) or down (CT_min_) at a rate of 0.25 °C min^−1^. This was repeated twice to yield sample sizes of *n* = 20 per treatment. A thermocouple (type T 36 SWG) connected to a digital thermometer (53/54IIB, Fluke Cooperation, USA) was inserted into the control chamber to monitor chamber temperature. The CT_max_ and CT_min_ were defined as the temperature at which each individual insect lost coordinated muscle function, which was considered as lack of response to mild prodding (e.g. [15]).

### 2.4 Supercooling points (SCPs)

Adult parasitoids (24–48 h old) SCPs were assayed as outlined by Nyamukondiwa et al. [10]. For each species, sixteen insects were individually placed in 0.65 ml microcentrifuge tubes where each insect was placed in contact with the tip of a type-T copper-constantan thermocouple (762–1121, Cambridge, UK). The thermocouples were inserted through the lid of each tube with both insect and thermocouple secured in place by a cotton wool. All thermocouple readings were taken via two 8-channel Picotech TC-08 (Pico Technology, Cambridge, UK) that relayed information to a computer equipped with PicoLog software for windows (Pico Technology, Cambridge, UK). Temperatures were continuously monitored and recorded at 1s intervals. In all cases, experiments commenced at a set point temperature of 15°C for 10 mins (to allow equilibration of insect body temperatures) before ramping down at 0.5°C min^-1^ until SCPs were recorded. In the current study, SCP for each individual was defined as the lowest temperature recorded prior to a spike in temperature associated with the latent heat of crystallization (see [10]).

### 2.5 Chill coma recovery time (CCRT)

For both species, CCRTs were assayed as outlined by Weldon et al. [42]. A total of 10 replicate adults (24–48 h old) were placed individually in 0.65ml microcentrifuge tubes and then loaded into a large zip-lock bag which was subsequently submerged into a water bath (Systronix, Scientific, South Africa). The water bath, filled with 1:1 water: propylene glycol mixture, was set at 0°C for 1 hour. After 1 hour at chill-coma temperature, the tubes were removed from the water bath and transferred to a Memmert climate chamber set at 28°C, 65% RH for recovery. The chamber was connected to a camera (HD Covert Network Camera, DS-2CD6412FWD-20, Hikvision Digital Technology Co., Ltd, China) that was linked to a computer where observations were recorded. CCRT was defined as the time (in minutes) required for an adult to stand upright on its legs [43]. This was repeated three times to yield sample sizes of *n* = 30 per treatment.

### 2.6 Heat knock down time (HKDT)

HKDTs for both species were assayed following Weldon et al. [42]. Ten replicate adult parasitoids (24 to 48 hrs) were individually placed in 0.65ml microcentrifuge tubes and placed in a climate chamber connected to a camera linked to a computer as in CCRT. The tubes carrying the parasitoids were then exposed to a test temperature of 45±0.3°C, 65% RH in the climate chamber. This knockdown temperature (45°C) was selected basing on preliminary investigations of upper critical temperatures to activity ranging 39.5±0.99°C and 44.6±0.63°C for *C*. *sesamiae* and *C*. *flavipes*, respectively. This was repeated three times to yield sample sizes of *n* = 30 for each species. All observations were made from the climate chamber video recording system. HKDT was defined as the time (in minutes) at which organisms lost activity following exposure to 45°C in the climate chamber.

### 2.7 Microclimate data recordings

Temperature data were recorded using Thermocron iButtons (Dallas Semiconductors, Model DS1920) (0.5°C accuracy; 1 h sampling frequency) from a maize field, Tswana Foods Farm, Glen Valley, Gaborone (S24.60213; E25.97820; 953m.a.s.l) during the period October 2015 to July 2016 to determine the thermal environment experienced by *C*. *sesamiae* and *C*. *flavipes* in the field. The field is known to host these two parasitoid species. iButtons were placed under a tree canopy (shaded environment), 1 m above the ground. This height reflects an environment where both congeneric *Cotesia* species and their stemborer hosts commonly ‘operate’.

### 2.8 Statistical analyses

Data analyses were carried out in STATISTICA, version 13.2 (Statsoft Inc., Tulsa, Oklahoma) and R version 3.3.0 [44]. LLT and ULT assays, SCPs, HKDT and CCRT results did not meet the assumptions of ANOVA, thus were analysed using generalized linear models (GLZ) assuming a binomial (LLT and ULT) and Gaussian (SCPs, HKDT and CCRT) distribution and a logit link function. This involved testing significance of temperature, duration of exposure and their interactions in both LLT and ULT assays, species versus SCPs, species versus recovery time for CCRT and species versus knockdown time for HKDT.

CTLs met the linear model assumptions of constant variance and normal errors, therefore, they were analysed using one-way ANOVA. In this case the categorical predictor was the developmental stage and the dependent variable was either CT_min_ or CT_max_. Tukey-Kramer’s *post-hoc* tests were used to separate statistically heterogeneous groups.

## 3. Results

### 3.1 Lower and upper lethal temperature assays

**Fig 1:**
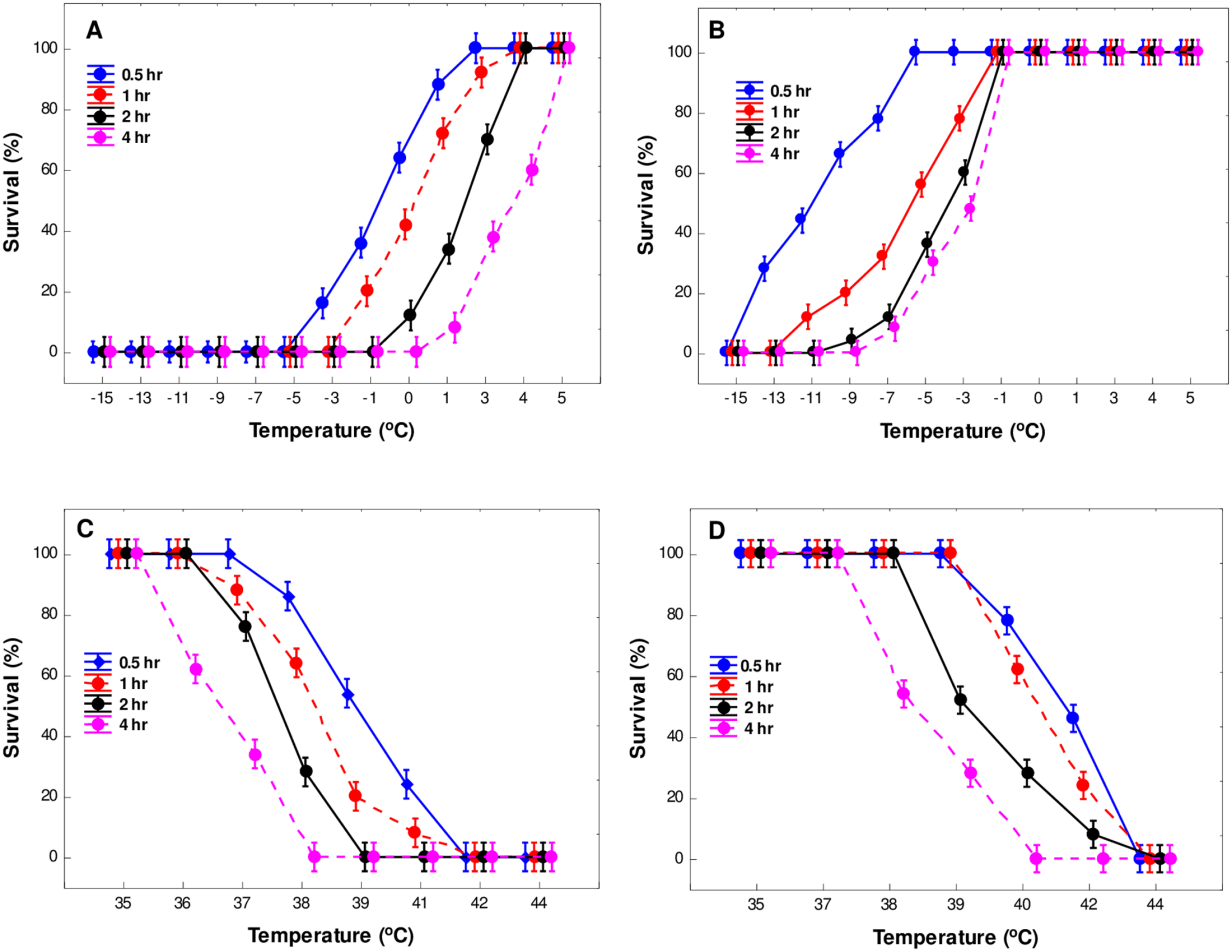
Mean (±95% confidence limit) survival of (A) *C*. *sesamiae* and (B) *C*. *flavipes* at different low temperatures, and (C) *C*. *sesamiae* and (D) *C*. *flavipes* at different high temperatures applied over four different durations. n = 50 per each temperature/time treatment.

Temperature and duration of exposure significantly influenced survival of *C*. *sesamiae* and *C*. *flavipes* adults at both low and high temperatures (*P* < 0.0001) (Table 1). An increase in severity of low and high temperature exposure resulted in increased mortalities for both species (Fig 1A–1D). Similarly, an increase in duration of exposure to lethal low and high temperatures generally resulted in survival decrease for both *C*. *sesamiae* and *C*. *flavipes* adults (Fig 1A–D). Duration of exposure × temperature interaction effects were highly significant for *C*. *flavipes* ULT (*P* < 0.001) whilst the same interactions were not significant (*P* > 0.05) for both species’ (LLT) and *C*. *sesamiae* (ULT) (Table 1). A comparison of 0.5, 1, 2 and 4 h durations for *C*. *sesamiae* and *C*. *flavipes* following exposure to lethal low temperatures ranged from -5 to 5°C and -15 to -1°C for 0–100% survival respectively (Fig 1A and 1B). Upper lethal temperatures for the same durations of exposure ranged from 35 to 42°C for *C*. *sesamiae* and 37 to 44°C for *C*. *flavipes* (Fig 1C and 1D).

**Table 1:** Summary statistical results of the effects of temperature, duration of exposure and their interactions on the survival of *Cotesia sesamiae* and *Cotesia flavipes* adults following lower and upper lethal temperature treatments. Analysis were done using generalized linear models (GLZ) assuming binomial distribution with a logit link function in R version 3.3.0.

Parameter	d.f	*χ*^2^	P-Value
**Lower Lethal Temperature**			
*Cotesia sesamiae*			
Duration	3	337.48	<0.0001
Temperature	7	1306.56	<0.0001
Temperature × Duration	21	26.47	0.1892
*Cotesia flavipes*			
Duration	3	358.91	<0.0001
Temperature	7	1061.1	<0.0001
Temperature × Duration	21	24.19	0.2838
**Upper Lethal Temperature**			
*Cotesia sesamiae*			
Duration	3	317.98	<0.0001
Temperature	6	1211.61	<0.0001
Temperature × Duration	18	23.15	0.185
*Cotesia flavipes*			
Duration	3	296.14	<0.0001
Temperature	5	977.14	<0.0001
Temperature × Duration	15	32.52	<0.001

### 3.2 Critical thermal limits (CTLs)

**Fig 2:**
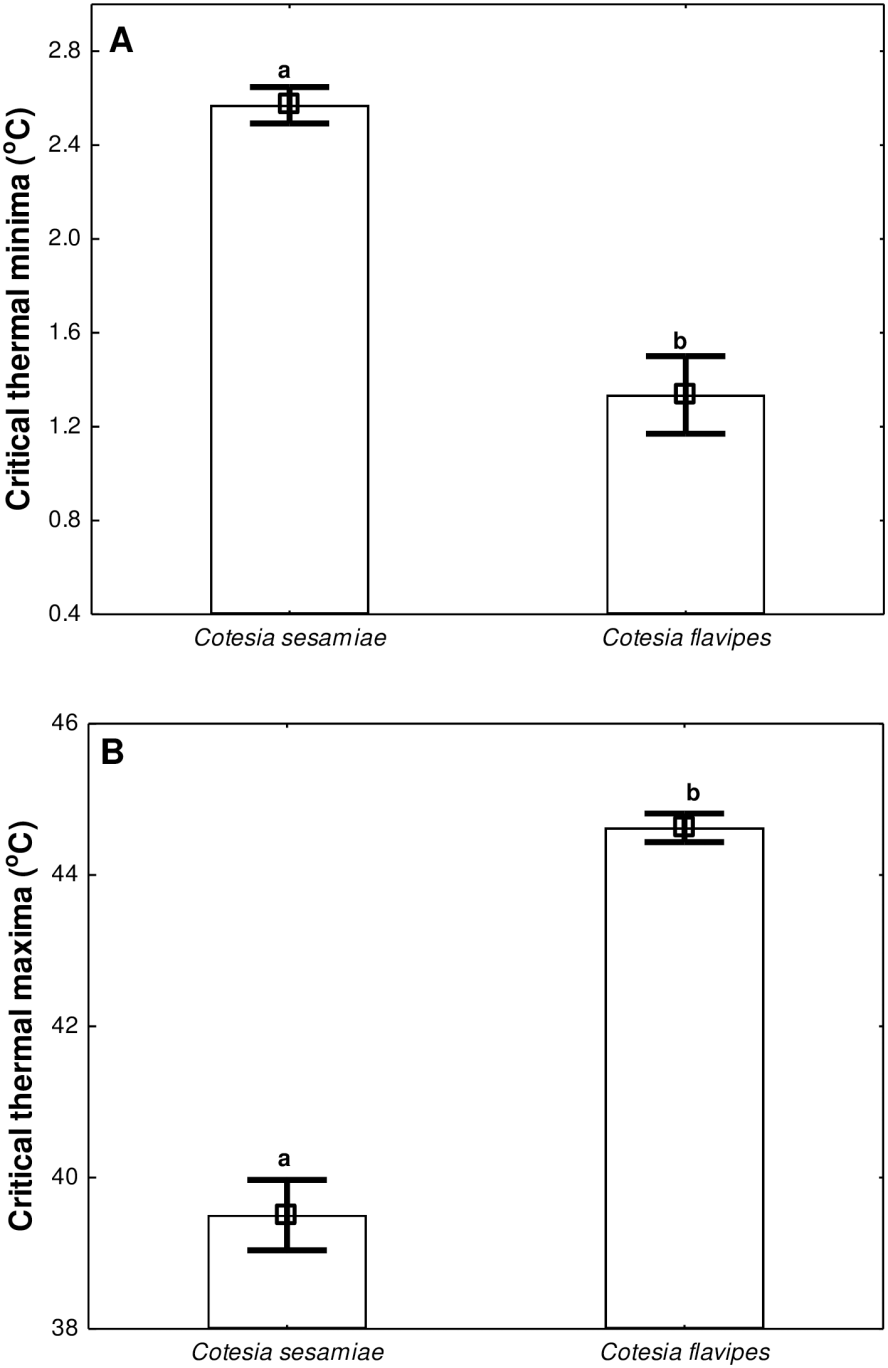
Effects of species (*Cotesia sesamiae* and *Cotesia flavipes*) on (A) Critical thermal minima and (B) Critical thermal maxima. Error bars represent 95% confidence limits (N = 20). Means with the same letter are not significantly different from each other.

Critical thermal minima and maxima varied significantly between *C*. *sesamiae* and *C*. *flavipes* adults (Table 2, Fig 2A and 2B). *Cotesia flavipes* recorded a significantly lower (*P* < 0.001) CT_min_ (1.34±0.59°C) than *C*. *sesamiae* (2.57±0.41°C). Similarly, *C*. *flavipes* also recorded a significantly higher (*P* < 0.001) CT_max_ (44.63±0.63°C) compared to congeneric *C*. *sesamiae* (39.51±0.99°C).

**Table 2:** Summary statistical results from full factorial ANOVA showing effects of species on critical thermal limits (CT_max_ and CT_min_).

Trait	Effect	SS	DF	MS	F	P
CT _min_	Intercept	152.49	1	152.49	2006.79	<0.0001
	Species	15.25	1	15.25	200.72	<0.0001
	Error	2.89	38	0.076		
CT _max_	Intercept	70778.57	1	70778.57	122885.1	< 0.001
	Species	262.14	1	262.14	455.1	< 0.001
	Error	21.89	38	0.58		

### 3.3 Supercooling points (SCPs)

**Fig 3:**
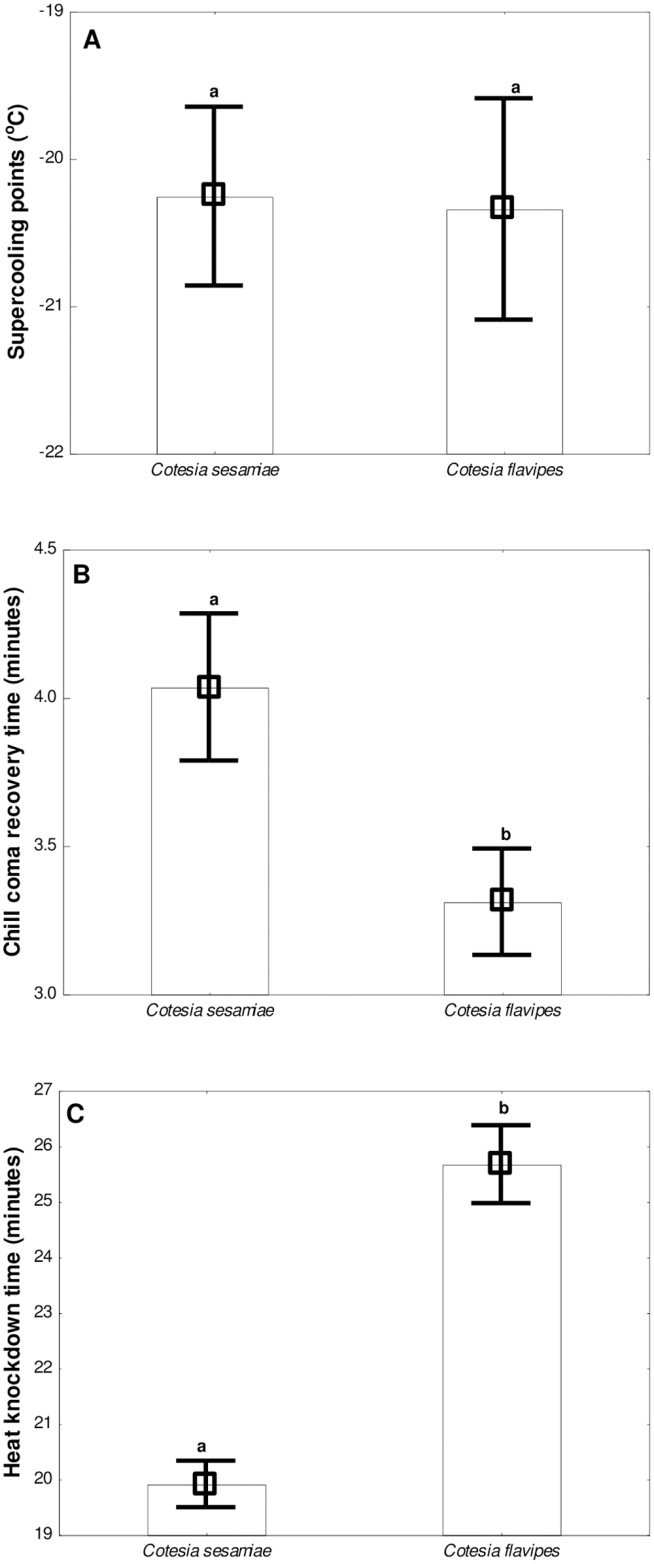
The effect of species (*Cotesia sesamiae* and *Cotesia flavipes*) on (A) supercooling points (N = 16 per group), (B) chill coma recovery time (at 0°C chill coma temperature) (N = 30 per group) and (C) heat knockdown time (at 45°C knockdown temperature) (N = 30 per group). Error bars represent 95% confidence limits. Means with the same letter are not significantly different from each other according to Tukey-Kramer’s *post-hoc* tests.

There was no significant difference in the supercooling points (*χ*^2^ = 0.0368, d.f = 1, *P* = 0.848) between *C*. *sesamiae* and *C*. *flavipes* (Fig 3A). The mean supercooling points for *C*. *sesamiae* and *C*. *flavipes* adults were -20.26±1.07°C and -20.34±1.19°C respectively (Fig 3A). SCPs were lower (Fig 3A) than the LLTs (Fig 1A and 1B) for both parasitoid species indicating that mortality temperatures were above those of supercooling.

### 3.4 Chill coma recovery time (CCRT)

There was a significant difference in CCRT between *C*. *sesamiae* and *C*. *flavipes* adults (*χ*^2^ = 23.41, d.f = 1, *P* < 0.0001) (Fig 3B). The average CCRTs were 3.31±0.69 and 4.01±0.82 minutes for *C*. *flavipes* and *C*. *sesamiae* adults respectively (Fig 3B). *Cotesia flavipes* recovered significantly faster than *C*. *sesamiae* following exposure to chill-coma temperature (0°C; 1 h) (Fig 3B), indicating a higher tolerance to low temperature for *C*. *flavipes* relative to *C*. *sesamiae*.

### 3.5 Heat knockdown time (HKDT)

Like in CCRT, there was a significant difference in HKDT between *C*. *sesamiae* and *C*. *flavipes* (*χ*^2^ = 207.77, d.f = 1, *P* < 0.0001) (Fig 3C). The mean HKDTs for *C*. *sesamiae* and *C*. *flavipes* adults were 19.91±1.06 and 25.67±1.37 minutes respectively (Fig 3C). *Cotesia flavipes* showed a significantly longer knock-down time than *C*. *sesamiae* following exposure to knock-down temperature (45.0±0.3°C) (Fig 3C), symbolising a significantly higher resistance to heat shock for the exotic *C*. *flavipes* relative to indigenous *C*. *sesamiae*.

### 3.6 Microclimate data recordings

**Fig 4:**
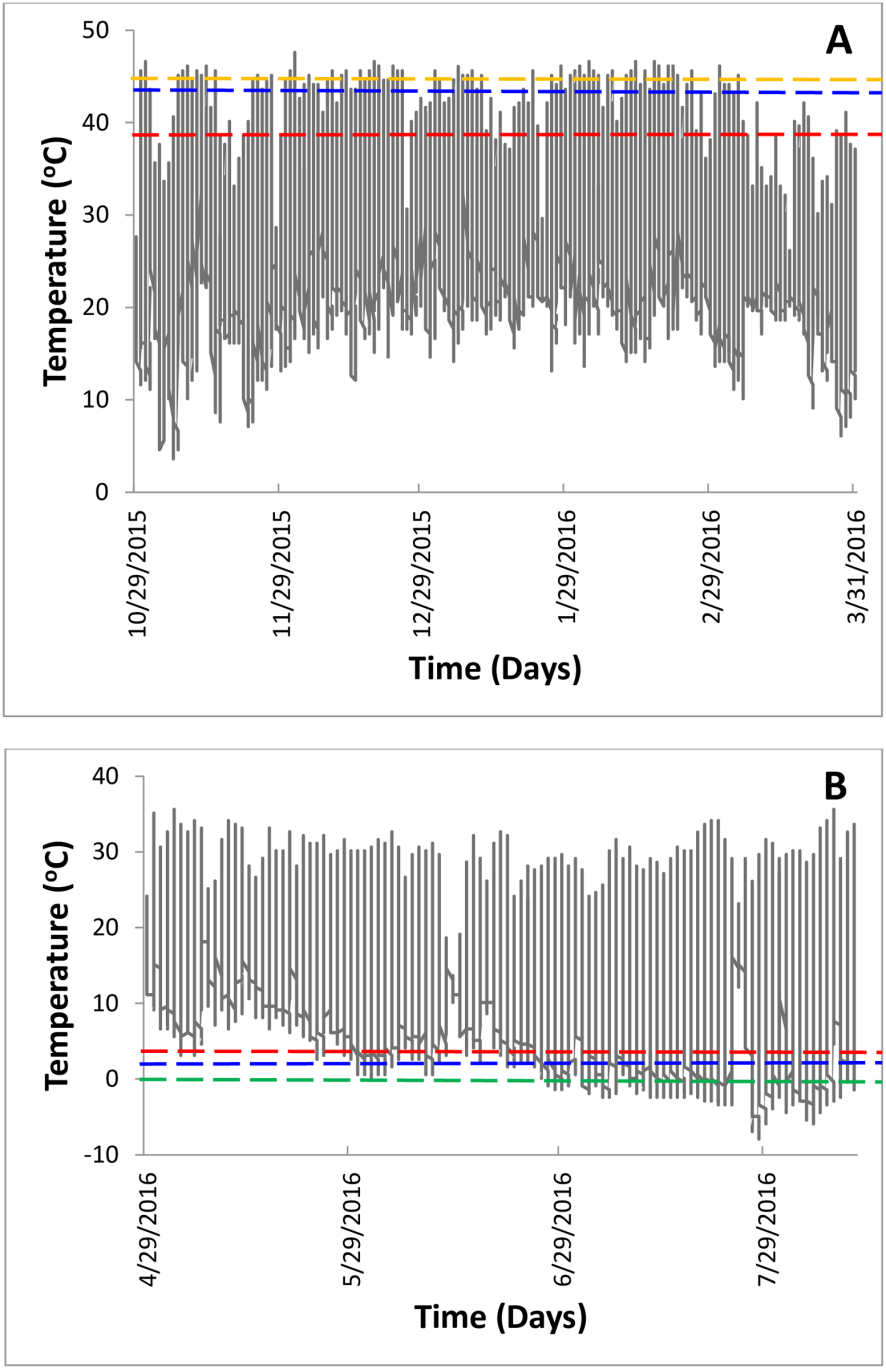
Microclimatic temperature data in (A) austral summer (October 2015 –March 2016) and (B) winter (April—July 2016) from a maize field, Tswana Foods Farm, Glen Valley, Gaborone, Botswana (S24.60213; E25.97820; 953m.a.s.l), hosting both *C*. *sesamiae* and *C*. *flavipes*. Blue and red dotted lines represent CT_max_ and CT_min_ for *C*. *flavipes* and *C*. *sesamiae* respectively whereas orange and green dotted lines represent heat knockdown and chill coma temperature respectively for both species.

Temperature recordings showed that shaded temperatures ranged from -7.9 to 46.6°C with low temperatures recorded during winter (April to July) and high temperatures during summer (October to March). A comparison of traits measured here (CTLs, CCRT and HKDT) versus records of microclimate temperature showed that these stressful thermal environments are often surpassed for both *C*. *flavipes* and *C*. *sesamiae* (Fig 4), albeit more so for *C*. *sesamiae* than *C*. *flavipes*.

## 4. Discussion

Evolutionary history, basal thermal tolerance and phenotypic plasticity mediate insect population phenologies and dynamics including biogeographic patterns and fitness traits under climate variability associated with anthropogenic climate change [45,46].

### 4.1 Lower and upper lethal temperature assays

In the current study, survival of *C*. *sesamiae* and *C*. *flavipes* was dependent on both severity and duration of temperature exposure as in other insect taxa [11,2,18]. We report *C*. *sesamiae* LLTs and ULTs ranging -5 to 5°C and 35 to 42°C respectively (for 0.5-4h exposures), in keeping with related studies (see [18]). This result affirms the significance of magnitude of temperature and duration of exposure on overall performance. At more extreme temperatures, duration of exposure was less important than more benign temperatures (see Fig 1A–1D), perhaps due to the irreversible damage on cell and protein function caused by extreme acute temperatures [47]. For 0.5–4 h treatments, LLTs for *C*. *sesamiae* and *C*. *flavipes* ranged from -5 to 5°C and -15 to -1°C whilst ULTs ranged from 35 to 42°C and 37 to 44°C respectively, suggesting that *C*. *flavipes* is more cold and heat tolerant than *C*. *sesamiae*. Given these responses, an important question will be whether these two species may continue coexisting in agroecosystems efficiently regulating lepidopteran stemborer populations under the current and projected climate change. Microclimatic data recorded in this study showed that thermal limits to activity and both low and high temperatures eliciting *C*. *sesamiae* and *C*. *flavipes* mortalities are frequently experienced under field conditions (Fig 4). However, the magnitude of these, appear to be more pronounced for *C*. *sesamiae* relative to *C*. *flavipes*. This therefore indicates that in the absence of compensatory mechanisms, physiological performance of these parasitoid species may be compromised. In consequence, this may negatively influence population phenologies and biogeography of these species under global change. With African temperatures projected to increase [48], at the same time associated with high frequency of heat waves, and cold snaps [33], it is highly likely that *C*. *flavipes* will survive more prolonged low and high temperature extremes than its congener *C*. *sesamiae*. When *C*. *partellus* is the host, *C*. *flavipes* has been reported to have a higher host searching efficiency even at low stemborer host densities than *C*. *sesamiae* [49]. Given these survival advantages, *C*. *flavipes* may, in all likelihood, have greater fitness potential than *C*. *sesamiae* in both cooler and warmer environments. Moreover, ULT and LLT temperatures reported here are often experienced in natural environments (see [18]) (see Fig 4), indicating the ecological significance of these findings. Furthermore, sudden heat waves and cold snaps are expected to rise with global change [29] and this likely has a compounding effect on fitness traits and survival of the two congeneric parasitoid species.

### 4.2 Critical thermal limits

The present study also demonstrated how temperature can limit the functional activity of both parasitoid species. CTLs are regarded as ecologically relevant measures of determining the impact of thermal variability on insect functional activities, e.g. mating, host searching, migration, feeding and others [50]. These activities represent key fitness traits for insects and in particular parasitoids, whose ecological functions depend on host searching ability [38], an activity trait highly dependent on temperature [6]. *Cotesia flavipes* had lower CT_min_ and higher CT_max_ compared to *C*. *sesamiae* (Fig 2), indicating a performance advantage for the exotic *C*. *flavipes* relative to congeneric *C*. *sesamiae* at both thermal extremes. This result also translates into a broader thermal activity window for *C*. *flavipes* relative to its congener (see Fig 4). As such, *C*. *flavipes* may likely optimise key life history activities better off than congeneric *C*. *sesamiae* under stressful and variable thermal regimes. Hence, under changing climates, biogeographic patterns and abundance of *C*. *sesamiae* will be more constrained than *C*. *flavipes* due to differential thermal activity windows. In other insect taxa, such declines in abundance and changes in biogeographic patterns have been attributed to changes in population dynamics due to the influence of temperature (see [34,51]). Hence, thermal tolerance superiority of *C*. *flavipes* reported here coupled with superior host searching ability [38], may likely make it a better competitor thus likely dominant over *C*. *sesamiae* in sub-Saharan Africa, and where the two share the same niche.

### 4.3 Supercooling points

We also document, for the first time, SCPs for adult *C*. *flavipes* and *C*. *sesamiae*. The average SCPs for *C*. *sesamiae* and *C*. *flavipes* were -20.26±1.14 and -20.34±1.41 respectively. Overwintering insects can be regarded as freeze tolerant (surviving ice formation within tissues), freeze intolerant (not surviving ice formation within tissues) [52] and chill susceptible (killed by chilling) [53]. Generally, insect species with depressed SCPs ranging between ~ -20°C to -30°C are regarded as freeze intolerant [54]. While our findings show both *C*. *sesamiae* and *C*. *flavipes* SCPs ~ -20°C, the failure to recover following supercooling suggest both species may be chill susceptible. Moreover, chill susceptibility is further affirmed by LLT results (Fig 1A and 1B) which indicated mortality temperatures for both species were way above those of supercooling. Nevertheless, supercooling ability remains a significant survival strategy for freeze intolerant species, likely facilitated by rapid accumulation of cryoprotectants and extracellular agents such as sugars, polyols and sorbitol, to avoid membrane rupture [53].

### 4.4 Chill coma recovery time

Chill coma recovery time is another important indicator of cold stress resistance in terrestrial arthropods [55]. Microclimate data also showed that chill coma temperatures are regularly encountered in the field (Fig 4B) during winter indicating that these parasitoid species may go into coma in their lifetime thus compromising their performance. In the present study, *C*. *flavipes* recovered significantly faster than *C*. *sesamiae* following exposure to chill coma temperature (0°C for 1 h), suggesting cold tolerance and survival advantage over its congener when faced with cold shock. Chill coma recovery has been reported to be mediated by rapid resumption of ion homeostasis [56]. Therefore, it is likely that the faster chill coma recovery for *C*. *flavipes* may be through enhanced ability for ion homeostasis relative to *C*. *sesamiae*. This is despite the two species having shown similar SCPs. Our study therefore also demonstrates the complexity of rapid thermal responses among insects and that different metrics of thermal tolerance may suggest divergent conclusions.

### 4.5 Heat knockdown time

*Cotesia flavipes* took more time (25.67±1.37 minutes) to be knocked down at high temperature (45°C) than *C*. *sesamiae* (19.91±1.06), further confirming its enhanced fitness advantage when faced with heat shock compared to *C*. *sesamiae*. These differential responses may be a result of the differences in the ability to rapidly express transient genetic responses responsible for upregulation of chaperone proteins that enable survival and activity at high temperatures [57]. In nature, microclimate data also revealed that *C*. *sesamiae* and *C*. *flavipes* experience knockdown temperature (see Fig 4A). Therefore, the ability to rapidly shorten or avoid heat knockdown and recover from chill coma temperatures is of high ecological relevance as any limited locomotion due to temperature stress, attributed to cessation of neuromuscular activity due to disruption of ion homeostasis [55], may lead to opportunistic predation by natural enemies. It is therefore highly likely that *C*. *sesamiae* may be more prone to such predation than *C*. *flavipes* under stressful thermal environments. Furthermore, prolonged inactivity following chill coma and heat knockdown may result in compromised realization of life history traits including reproductive fitness and ultimately delivery of the ecosystem services like host pest parasitisation. Thus, our results also report a fitness and survival advantage of exotic *C*. *flavipes* relative to indigenous *C*. *sesamiae* following heat shock.

### 4.6 Conclusion

Overall, our results reveal *C*. *flavipes* has superior basal thermal tolerance than congeneric *C*. *sesamiae* at both thermal extremes. Even though *C*. *sesamiae* and *C*. *flavipes* can coexist with interactive synergistic effects that maximize biocontrol efficacy against lepidopteran stemborer species [29,22], we conclude that *C*. *flavipes* may become dominant under rapidly changing environments due to its superior thermal physiology. This differential thermal tolerance may negatively impact on the parasitoids ecological function hence interruption of host-parasitoid relationships. In consequence, this may lead to an increase in host outbreaks thus affecting household and national food security. However, we make this conclusion with caveats as we only determined basal responses and not phenotypic plasticity of the various matrices, host thermal responses, quiescence or diapause which also have a bearing on parasitoid matching. Other studies have shown how several factors such as within- and transgenerational -thermal history [15], age and feeding status [8,16] and ontogeny [49] can influence responses to thermal exposure with significant potential for mitigating adverse effects of climate change (reviewed in [58]. Future studies should therefore endeavor to explore such plasticity since the knowledge can aid enhancement of quality of mass-reared parasitoid insects [59]. For example, developmental and adult acclimation in moths resulted in improved field performance of mass- reared *Cydia pomonella* (measured as flight response to pheromone traps) under conditions similar to acclimation conditions [11]. This evidence is in addition to earlier work on the Queensland fruit fly where manipulation of thermal plasticity through preconditioning resulted in improved field performance [59]. Pest managers can therefore utilize such preconditioning to improve the survival and performance of the parasitoids and augment natural populations, in particular for *C*. *sesamiae* which had a poor basal thermal tolerance. This will not only aid performance of *C*. *sesamiae*, but also ensure that introduced alien *C*. *flavipes* may not become over dominant to ensure maintenance of native biodiversity in agroecosystems.
